# DCMS: A data analytics and management system for molecular simulation

**DOI:** 10.1186/s40537-014-0009-5

**Published:** 2014-11-26

**Authors:** Anand Kumar, Vladimir Grupcev, Meryem Berrada, Joseph C Fogarty, Yi-Cheng Tu, Xingquan Zhu, Sagar A Pandit, Yuni Xia

**Affiliations:** Department of Computer Science and Engineering, University of South Florida, 4202 E. Fowler Ave., ENB118, Tampa, 33620 Florida USA; Department of Physics, University of South Florida, 4202 E. Fowler Ave., PHY114, Tampa, 33620 Florida USA; Department of Electrical Engineering and Computer Science, Florida Atlantic University, 777 Glades Road, EE308, Boca Raton, 33431 Florida USA; Department of Computer Science, Indiana University - Purdue University Indianapolis, 723 W. Michigan St, SL280E, Indianapolis, 46202 Indiana USA

**Keywords:** Scientific database, Molecular simulation, Molecular dynamics, Data compression, Spatiotemporal database

## Abstract

Molecular Simulation (MS) is a powerful tool for studying physical/chemical features of large systems and has seen applications in many scientific and engineering domains. During the simulation process, the experiments generate a very large number of atoms and intend to observe their spatial and temporal relationships for scientific analysis. The sheer data volumes and their intensive interactions impose significant challenges for data accessing, managing, and analysis. To date, existing MS software systems fall short on storage and handling of MS data, mainly because of the missing of a platform to support applications that involve intensive data access and analytical process. In this paper, we present the database-centric molecular simulation (DCMS) system our team developed in the past few years. The main idea behind DCMS is to store MS data in a relational database management system (DBMS) to take advantage of the declarative query interface (*i.e.*, SQL), data access methods, query processing, and optimization mechanisms of modern DBMSs. A unique challenge is to handle the analytical queries that are often compute-intensive. For that, we developed novel indexing and query processing strategies (including algorithms running on modern co-processors) as integrated components of the DBMS. As a result, researchers can upload and analyze their data using efficient functions implemented inside the DBMS. Index structures are generated to store analysis results that may be interesting to other users, so that the results are readily available without duplicating the analysis. We have developed a prototype of DCMS based on the PostgreSQL system and experiments using real MS data and workload show that DCMS significantly outperforms existing MS software systems. We also used it as a platform to test other data management issues such as security and compression.

## Background

Recent advancement in computing and networking technologies has witnessed the rising and flourishing of data-intensive applications that severely challenge the existing data management and computing systems. In a narrow sense, data-intensive applications commonly require significant storage space and intensive computing power. The demand of such resources alone, however, is not the only fundamental challenge of dealing with big data [1-3]. Instead, the complications of big data are mainly driven by the complexity and the variety of the data generated from different domains [4]. For example, online social media has now been popularly used to collect real-time public feedback related to specific topics or products [5]. Data storage and management systems should support high throughput data access with millions of tweets generated each second [4]. Meanwhile, the tweets may be generated from different geographic regions, using different languages, and many of them may contain spam messages, typos, and malicious links etc. In addition to the low level data cleansing, access, and management issues, user privacy and public policies should also be considered (and integrated) in the analytical process for meaningful outcomes.

For many other application domains, such as scientific data analysis, the above big data complications also commonly exist. For example, particle simulation is a major computational method in many scientific and engineering fields for studying physical/chemical features of natural systems. In such simulations, a system of interest is treated as a collection of potentially large number of particles (*e.g.*, atoms, stars) that interact under classical physics rules. In the molecular and structural biology world, such simulations are generally called Molecular Simulations (MS). By providing a model description for biochemical and biophysical processes at a nanoscopic scale, MS is a powerful tool towards fundamental understanding of biological systems.

**Figure 1:**
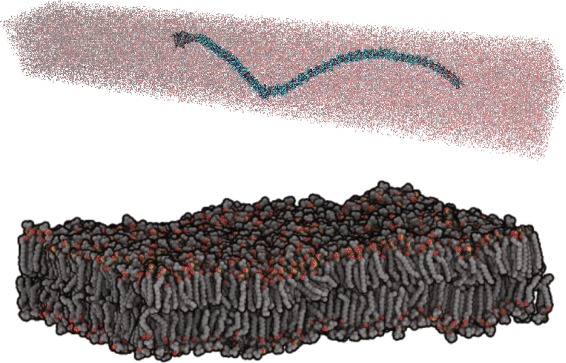
**Snapshots of two MS systems: a collagen fiber structure with 890,000 atoms (top) and a dipalmitoylphosphatidylcholine (DPPC) bi-layer lipid system with 402,400 atoms (bottom).**

At present time, the field of MS has a handful of software systems employing their proprietary or open formats for data storage [6-8]. Although many of them are carefully designed to achieve maximum computational performance in simulation, they significantly fall short on storage and handling of the large scale data output. The MS by their nature generate a large amount of data in a streaming fashion - a system could consist of millions of atoms and one single simulation can easily run for tens of thousands of time steps. Figure 1 shows two (small) examples of such simulations. One salient problem of existing systems is the lack of efficient data retrieval and analytical query processing mechanisms.

In this paper, we present our recent research efforts in advancing big data analytic and management systems for scientific simulation domains, which usually generate large datasets with temporal and spatial correlations for analysis. Our research mainly emphasizes on the design of the data management system in supporting intensive data access, query processing, and optimization mechanisms for MS data. The main objective of our study is to produce high performance techniques for the MS community to accelerate the discovery process in biological/medical research. In particular, we introduce the design and development of a Database-Centric Molecular Simulation (DCMS) framework that allows scientists to efficiently retrieve, query, analyze, and share MS data.

A unique feature of DCMS is to build the system framework on top of a relational database management system (RDBMS). Such a decision is justified by careful analysis of the data processing requirements of the target application: since MS data is spatiotemporal in nature, existing DBMS provides significant advantages in modeling and application development. Plus, we can leverage the results of decades of research in spatiotemporal databases that are often (at least partially) implemented in RDBMSs. On the other hand, the unique features of MS data analysis/querying workload call for significant improvement and new functionalities in existing RDBMSs. A salient problem we face is the high computational complexity in processing analytical queries that are not seen in typical databases, demonstrating another dimension of difficulty shared by many of today’s big data applications. For MS, there are also data compression and data security issues that require innovative solutions. Therefore, our work in DCMS focuses on meeting those challenges by augmenting the DBMS kernel with novel data structures and query processing/optimization algorithms. As a result, our system achieves significant improvement in data processing efficiency as compared to legacy solutions.

### Related work

In current MS software [9-11], simulation data is typically stored in data files, which are further organized into various levels of directories. Data access is enabled by encoding the descriptions of the content in files into the names of files and directories, or storing more detailed descriptions about the file content in separate *metadata* files. Under the traditional file-based scheme, data/information sharing among MS community involves shipping the raw data packed in files along with the required format information and analysis tools. Due to the sheer volume of MS data, such sharing is extremely difficult, if possible at all. Two MS data analysis projects, BioSimGrid [12] and SimDB [13], store data and perform analysis at the same computer system and allow users remotely send in queries and get back results. This approach is based on the premises that: (1) analysis of MS data involves projection and/or reduction of data to smaller volume; (2) users need to exchange the reduced representation of data, rather than the whole raw data. In a similar project [14], databases are used to store digital movies generated from visualization of MS datasets.

In BioSimGrid and SimDB, relational databases are used to store and manage the metadata information. However, both systems *store raw MS data as flat files instead of database records*. Thus, the database only helps in locating the files of interest by querying the metadata. Further data retrieval and analysis are performed by opening and scanning the files located. Such an approach suffers from the following drawbacks: (1) *Difficulties in the development and maintenance of application programs*. Specific programs have to be coded for each specific type of queries using a general-purpose programming language such as C. This creates high demand for experienced programmers and thus limits the type of queries the system can support. (2) *Lack of a systematic scheme for efficient data retrieval and analysis.* An operating system views data as continuous bytes and only provides simple data access interfaces such as *seek* (i.e., jumping to a specific position of the file). Without data structures that semantically organize data records, data retrieval is often accomplished by sequentially scanning all relevant files. There is also a lack of efficient algorithms for processing queries that are often analytical in nature - most of existing algorithms are brute-force solutions. (3) *Other issues* such as data security and data compression are not sufficiently addressed.

The MDDB system [8] is close in spirit to DCMS. However, it focuses on data exploration and analysis within the simulation process rather than post-simulation data management. Another project named Dynameomics [15] coincided with the development of DCMS and delivered a database containing data from 11,000 protein simulations. Note that the main objective of the DCMS project is to provide a systematic solution to the problems mentioned above. To that end, most of our work is done within the kernel space of an open-source DBMS. In contrast to that, Dynameomics uses a commercial DBMS in its current form and attempts to optimize data management tasks at the application layer. We believe the DCMS approach has significant advantages in solving the last two issues mentioned above.

## Case description

### Issues

Here we summarize the data management challenges in typical MS applications.

**MS Data** A typical simulation outputs multiple *trajectory files* containing a number of snapshots (named *frames*) of the simulated system. Depending on the software and format, such data may be stored in binary form and undergo simple lossless compression. The main part of the data is very similar to those found in spatio-temporal databases. A typical trajectory file has some global data, which is used to identify the simulation, and a set of frames arranged in a sequential manner. Each frame may contain data entries that are independent of the atom index. The main part of trajectory frame is a sequential list of atoms with their positions, velocities, perhaps forces, masses, and types. These entries may contain additional quantities like identifiers to place an atom in particular residue or molecule. In file-based approach, the bond structure of residues is stored separately in *topology* files and the control parameters of a simulation are kept separately in *control* files. Hence, any sharing of data or analysis requires consistent exchange or availability of three types of files. Further complications in data exchange/use is due to different naming and storage convention used by individual researchers.

**MS Queries** Unlike traditional DBMSs where data retrieval is the main task, the mainstream queries in DCMS are analytical in nature. In general, an analytical query in MS is a mathematical function that maps the readings of a group of atoms to a scalar, vector, a matrix, or a data cube [13]. For the purpose of studying the statistical feature of the system, popular queries in this category include density, first-order statistics, second-order statistics, and histograms. Conceptually, to process such queries, we first need to retrieve the group of atoms of interest, and then compute the mathematical function. Current MS analysis toolboxes [6,7,9,11] accomplish these steps in an (algorithmically) straightforward way. Some of the analytical queries are computationally expensive. Popular queries can be found in Table 1 and we will elaborate more on those in Section “Analytical queries in DCMS”. Many types of analytical queries are unique to the MS field, especially those that require the counting of all *n*-body interactions (thus named *n-body correlation functions*). For example, the spatial distance histogram (SDH) is a 2-body correlation function in which all pairwise distances are to be counted. The query processing engines in traditional DBMSs are designed with only simple aggregate queries in mind. Therefore, one major challenge of this project is to design mechanisms for efficient processing of analytical queries in MS that go far beyond simple aggregates.

**Table 1:** **Popular analytical queries in MS**

**Query name**	**Definition/Description**
Moment of inertia	$I = \sum \limits _{i=1}^{n} m_{i}\mathbf {r}_{i} $
Moment of inertia on *z* axis	$\begin {array} {lcl} I_{z} & = & \sum \limits _{i=1}^{n} m_{i}r_{\textit {zi}} \end {array}$
Sum of masses	$\begin {array} {lcl} M & = & \sum \limits _{i=1}^{n} m_{i} \end {array}$
Center of mass	$\begin {array} {lcl} C & = & \frac {I}{M} \end {array}$
Radius of gyration	$\begin {array} {lcl} RG & = & \sqrt {\frac {I_{z}}{M}} \end {array}$
Dipole moment	$\begin {array} {lcl} D & = & \sum \limits _{i=1}^{n} q_{i}\mathbf {r}_{i} \end {array}$
Dipole histogram	$\begin {array} {lcl} D_{z} & = & \sum \limits _{i=1}^{n} \frac {D}{z} \end {array}$
Electron density	$\begin {array} {lcl} ED = \frac {\sum \limits _{i=1}^{n} (e_{i}-q_{i})} {dz*x*y} \end {array}$
Heat capacity	$ \frac {3000\sqrt {T}*boltz}{2*\sqrt {T}-n*df*VarT}$
Isothermal compressibility	$ \frac {VarV}{V_{\textit {avg}}*boltz*T*PresFac}$
Mean square displacement	$\begin {array} {lcl} msd & = & \langle (r_{t+\Delta _{t}}-r_{t}) \rangle \end {array}$
Diffusion constant	$\begin {array} {lcl} D_{t} & = & \frac {6*msd(t)}{t} \end {array}$
Velocity autocorrelation	$\begin {array} {lcl} V_{\textit {acor}} & =&\langle (V_{t+\Delta _{t}}*V_{t}) \rangle \end {array}$
Force autocorrelation	$\begin {array} {lcl} F_{\textit {acor}} & = & \langle (F_{t+\Delta _{t}} * F_{t}) \rangle \end {array}$
Density function	Histogram of atom counts
Spatial distance histogram (SDH)	Histogram of all atom-to-atom distances
RDF	$\begin {array} {lcl} rdf(r) & = & \frac {SDH(r)}{4*\pi *r^{2}*\sigma _{r}*\rho } \end {array}$

While analytical queries are the workhorse tools for scientific discovery, many require retrieval of data as the first step. Furthermore, visualization tools also interact with the database retrieving subsets of the data points. By studying the data access patterns of the analytical queries and such tools, we identify the following data access queries that are relevant in DCMS:

- *Point queries* are equivalent to accessing a single point at the 3D space, e.g., find the location and/or other physical measurements of an atom at a specific time frame. Such queries are extremely useful for many visualization tools. A typical scenario is: the visualization tool asks for a sample of *n* data points within a specific region that reflects the underlying distribution. This is done by issuing queries with randomly generated atom IDs.
- *Trajectory queries* retrieve all data points by fixing the value in one dimension. Two queries in this category are very popular in MS analysis: (1) single-atom trajectory (TRJ) query that retrieves the readings of a specific atom along time, and (2) frame (FRM) query that asks for the readings of all atoms in a specific time frame. These two queries, especially the second, are often issued to retrieve data points for various analytical queries such as the diffusion coefficient, in which we compute the root mean square displacement of all atoms in a frame.
- *Range (RNG) queries* are generalized trajectory queries with range predicates on one or more dimensions. For example, find all atoms in a specific region of the simulated space, or, find all atoms with velocity greater than 50 and smaller than 75. Range queries are the main building blocks of many analytical queries and visualization tasks.
- *Nearest neighbor (NN) queries* ask for the point(s) in a multidimensional space that are closest to a given point. For example, *retrieve the 20 closest atoms to a given iron atom*. This may help us locate unique structural features, e.g., certain part of the protein where a metal ion is bound to.

### DCMS architecture

**Figure 2:**
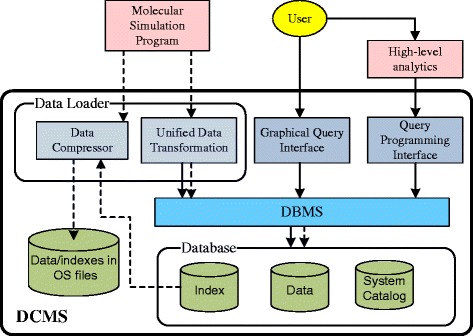
**The DCMS architecture.**

The architecture of the DCMS framework is illustrated in Figure 2, where the solid lines represent command flow and dotted lines represent data flow. At the core of DCMS is an integrated database system, including simulation parameters/states, simulation output data, and metadata for efficient data retrieval. An important design goal of DCMS is to allow scientists to easily transfer, interrogate, visualize, and hypothesize from integrated information obtained from a user-friendly interface as opposed to dealing with raw simulation data. To that end, DCMS provides various user interfaces for data input and query processing.

**Data loader** The data loader is responsible for transforming simulation data to the format required for storage in the database system. First, it can read and understand data generated by current simulation programs and stored in popular MS file formats (e.g., GROMACS, PDB). We developed, as a part of the data loader, a *unified data transformation module* to perform such translations. A user only needs to specify the file format his/her data follows and data loading will be performed automatically. Second, the raw data will also be sent to a *Data Compression* module to generate a volume-reduced copy to be stored as compressed files. The rationale of this design is to enable efficient transmission of MS data among different data centers where DCMS are deployed. We will address this issue in Section “Data compression”.

**User interfaces** Runtime data access in DCMS is provided by a graphical query interface and an SQL-based query programming interface. In DCMS, we envision two types of user programs: *primary queries* and *high-level data analytics*. The primary queries correspond to (raw) data retrieval queries that can be directly supported by the DBMS via SQL. Analytical queries are those containing application-specific logic and are directly used by scientists for scientific discovery. The latter builds upon query results of the primary queries. To ease the development of high-level analytics, an important design of DCMS is that the query interfaces are extensible: an analytical query written by a user (called *user-programmed analytics*) can be integrated into the current DCMS query interface (and become part of the *DCMS built-in analytics* that can be directly accessed by an SQL statement). By this, the code of analytical queries can be reused by other users to issue the same query or build new analytical queries based on the current ones.

In addition to the query programming interface, all built-in analytics and primary queries can also be accessed from a graphical query interface, which accepts user query specifications via web forms and translates such specifications into SQL statements. The main purpose of designing a graphical interface is to, again, ease the use of DCMS to an extent that users can perform data analysis without writing programs.

**Figure 3:**
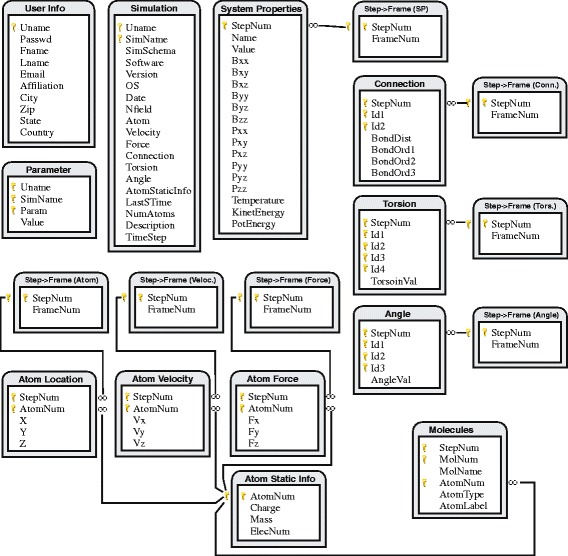
**Schema of the DCMS database.** The golden key symbol marks the primary keys and the lines represent foreign keys. Note that there exist foreign keys from all Ids in tables *Connection*, *Torsion*, and *Angle* to the *Atom Static Info* table. We did not draw them due to space limit.

**MS data modeling and database design** Generally, an MS dataset contains a small number of physical features of a large number of atoms recorded at different steps of the simulation. Some of the important features include 3D locations, velocity, charge, and forces. All data collected in one time interval is called a *frame*. The main body of an MS dataset is thus a collection of data items, each of which holds the information about an individual atom at a specific time frame. Conceptually, each data item can be viewed as a point in a multidimensional space with the dimensions representing the physical features we record. As a result, it fits the relational data model very well and we show the schemas corresponding to one simulation dataset in Figure 3. Note that an MS software such as GROMACS generates files holding information in rows. Naturally, our database design process started from a one-to-one mapping from those files to relations. We then refined the initial schemas by removing redundancy and reached a design shown in Figure 3.

First group of relations (i.e., *Simulation* and *Parameter*) describe time-invariant information of the simulation system. The *System Properties* table contains time-variant information of the entire system (instead of individual atoms). The *Atom Static Info* table holds the static features of an atom and forms a star-shaped schema with a series of other tables: *Location*, *Velocity*, *Force*, *Connection*, *Torsion*, *Angle*, and *Molecules*. The first three form the main body of the database - they represent atom states that change over time during the simulation. The reason why we cannot combine these three into one table is: MS programs usually do not output data at every step of the simulation, and different intervals (in steps) can be set to output location, velocity, and force. For the same reason, each of the three tables is linked to another table that maps the step number to the frame number. The next three tables hold information that describes atom to atom relationships. For example, a row in *Connection* represents a chemical bond between two atoms. Such relationships are time-variant therefore we again need to map their step numbers to frame numbers. The *Molecules* table is similar except it holds static relationship among the atoms. Specifically, each row in this table records the membership of one atom as a part of a molecule.

**Analytical queries in DCMS** As mentioned earlier, DCMS provides system support for analytical queries that are unique in MS. The most popular DCMS built-in analytics are listed in Table 1, in which we assume an MS system has *n* atoms, and denote the mass, coordinates, charge, and number of electrons of an atom *i* as *m*_*i*_, **r**_*i*_, *q*_*i*_, and *e*_*i*_, respectively. Roughly, such queries can be divided into two categories: (1)*One-body functions.* In computing this type of functions, the readings of each atom in the system is processed constant number of times, giving rise to *O*(*n*) total running time. All queries in Table 1 except the last two fall into this category. Most such queries are defined within a single frame while the various autocorrelation functions are defined over two different frames; (2) *Multi-body functions.* The computation of these functions requires interactions of all atom pairs (2-body) or triplets (3-body). Popular examples include Radial Distribution Function (RDF) [16-18] and some quantities related to chemical shifts [19]. These functions are often computed as histograms. For example, RDF is generally derived from a histogram of all atom-to-atom distances (i.e., Spatial Distance Histogram (SDH)). Straightforward computation of multi-body functions is very expensive therefore it imposes great challenges to query processing.

### Query processing and optimization in DCMS

In DCMS, we could rely on a legacy DBMS (e.g., PostgreSQL) to provide query processing mechanisms. However, we believe that explorations on the algorithmic issues in major DBMS modules (e.g., query processing and access methods) will further improve the efficiency of data analysis in DCMS. This is because existing DBMSs, with general-purpose data management as the main purpose, have little consideration of the *query types* and *user access patterns* that are unique in MS data analysis.

#### Primary queries

Data indexing is the most important database technique to improve the efficiency of data retrieval. In DCMS, algorithms for processing primary queries will be exclusively index-based to reduce data access time. To support a rich set of queries, multiple indexes are necessary. However, it is infeasible to maintain excessive number of indexes due to the extremely high storage cost for MS data. Note that MS databases are most likely read-only therefore the maintenance (update) cost of indexes can be ignored. We have designed and tested several novel indexes to handle the various queries in DCMS but finally adopted the following indexes in our implemented system: (1) the B ^+^-tree and a bitmap-based index which are the default indexes provided by PostgreSQL - they provide a certain level of support for some of the MS queries; and (2) a new index named Time-Parameterized Spatial (TPS) tree to provide further performance boost. We accordingly modify the query optimizer of the DBMS to generate query execution plans that take advantage of the aforementioned indexes.

##### TPS tree

For spatial (range, nearest neighbor, or spatial join) queries, Quadtree, R*-tree or SS-tree are the most popular indexes. The main challenge in DCMS comes from the continuous queries where time serves as an extra dimension therefore the above data structures are not suitable for MS queries. The design of TPS tree can be briefly described as *building a spatial index for each time frame in the dataset*. Then we need to combine neighboring trees to save space, taking advantage of the similarities among these trees. We decided to use Quadtree as the underlying spatial index to build TPS. This is because: (1) the performance of Quadtree in handling spatial queries is equivalent (sometimes even superior) to that of the R*-tree [20]; (2) the chances of getting an unbalanced tree (which is the main drawback of the Quadtree) are small due to the “spread-out" feature of MS data; and most importantly, (3) Quadtrees can be augmented to build other data structures needed for our high-level analytical query processing (Section “Analytical queries”).

A major challenge in designing the TPS tree is to *minimize the storage cost*. Our main idea is to share nodes among neighboring Quadtrees corresponding to consecutive time frames - similar to the historical R-tree (HR-tree) [21]. The node sharing in an HR-tree depends on the assumption that some objects do not move for a period of time, which is not applicable to MS data where all atoms move at all times. However, we can exploit the existence of slow-moving atoms or those that move together to achieve node sharing. To build a TPS tree, we start by creating a spatial tree for each time frame using bulk loading; and then merge nodes in neighboring trees iteratively.

#### Analytical queries

A straightforward view of analytical query processing consists of two stages: retrieve the raw data and compute the results in a separate program. While indexing techniques shown in Section “Primary queries” can speed up the first stage, further optimizations are made in DCMS by *pushing the computation into index construction*. To be more specific, we cache critical statistics of all atoms contained in a node of the TPS tree. Query results can be derived directly from such statistics, saving much time for visiting the raw data points. In the following text, we sketch our caching-based query processing strategy by visiting two groups of analytical queries.

##### One-body functions

Most one-body functions are *algebraic functions* [22] with features suitable for caching-based aggregation. Given a spatial region **A** in the simulated space, let us divide it into two disjoint subregions **A**_1_,**A**_2_. If we know relevant quantities for the computation a one-body function *f* of all atoms in both subregions, then the value of *f*(**A**) could be computed from those subregional quantities in constant time for most types of one-body functions. Take the center of mass (quantity *C* in Table 1) as an example: if we cache the values of the moment of inertia *I* and sum of mass *M* for both subregions, the mass center of region **A** can be computed by:

$$\frac{\sum_{i \in \mathbf{A}} m_{i} \mathbf{r}_{i}}{\sum_{i \in \mathbf{A}} m_{i}} = \frac{\sum_{i \in \mathbf{A}_{1}} m_{i} \mathbf{r}_{i} + \sum_{i \in \mathbf{A}_{2}} m_{i} \mathbf{r}_{i}}{\sum_{i \in \mathbf{A}_{1}} m_{i} + \sum_{i \in \mathbf{A}_{2}} m_{i}}\ = \frac{I_{\mathbf{A}_{1}}+I_{\mathbf{A}_{2}}}{M_{\mathbf{A}_{2}} + M_{\mathbf{A}_{2}}} $$

**Figure 4:**
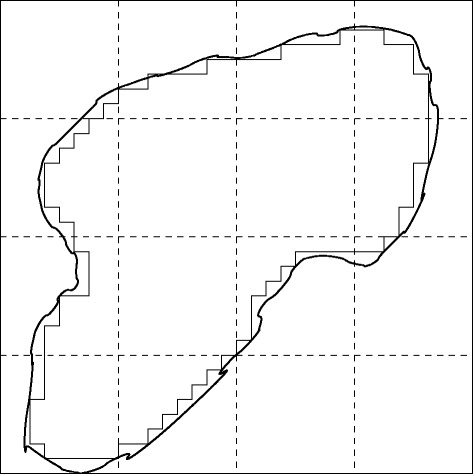
**A 2D illustration of an irregular query region.** Thin lines represent inclusive tree nodes visited at the 5th level in the Quadtree (the level 0 node in the tree covers the whole region).

With the above reasoning, we can cache the chosen quantities in all nodes of a TPS tree without increasing the time complexity of tree construction. To compute the *C* value in an arbitrary region (Figure 4), we can traverse the tree and build *C* incrementally from the cached values of all nodes that are totally included in the query region. Thus, the running time depends on the number of tree nodes visited (instead of *n*). In fact, it is easy to see that the time complexity is $O\left (v^{\frac 2 3} \right)$ where *v* is the volume of the query region. The savings on I/O time are also significant: cached quantities in a tree node can be orders of magnitude smaller (in size) than that of the raw MS data it covers [13].

##### Multi-body functions

The multi-body functions are all *holistic functions* [22] therefore cannot be computed in the same way as one-body functions. Current MS software adopts simple yet naïve algorithms to compute the multi-body functions [9]. For example, the SDH is computed by retrieving the locations of all atoms, computing all pairwise distances, and grouping them into the histogram - a *O*(*n*^2^) approach. For a large simulation system where *n* is big, this algorithm could be intolerably slow. In DCMS, we invested much efforts into algorithmic design related to such queries.

Our strategy for fast multi-body function computation follows the same path of the caching-based query processing. Specifically, we cache the *atom counts* in each spatial tree node and compute the multi-body functions based on these counts, avoiding the computation of pairwise interactions. Again, we do this on the TPS tree and call the atom counts of all nodes on one tree level a *density map* (which is basically the result of a *density function* query shown in Table 1). The main issue is how to translate the atom counts into a histogram. In the following text, we will sketch a series of algorithms we developed for SDH.

**Figure 5:**
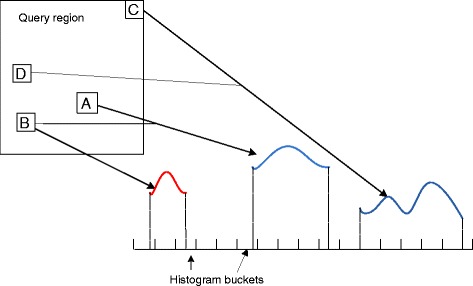
**Intuition behind computation of SDH by considering pairs of nodes in a density function.** Curves represent distribution of distances between two nodes (blue) or within a single node (red).

The main idea is to work on clusters of atoms (e.g., A, B, C, D shown in Figure 5) instead of individual atoms in deriving the function results. Beginning at a predetermined level, the TPS tree is traversed to see if any pair of nodes can form a distance range that is fully contained in a bucket of the histogram. This is the key operation in this algorithm and it is named *node resolving*. If a pair of nodes do not resolve, we keep resolving their child nodes in the tree. At leaf level, point-to-point distances between particles are computed to finish histogram processing. Such density-based algorithm achieves running time of $O \left (N^{\frac {5}{3}}\right)$ [23].

In many cases approximate query results are acceptable to users, therefore we developed approximate algorithms with much better performance to solve the SDH problem. Basically, we modified the node resolving operation to generate partial SDH faster. Whenever a desired error bound is reached while traversing the tree, the distance distribution into histogram buckets is approximated using certain heuristics. Such heuristics are constant time operations while having guaranteed error bound. Total running time of the algorithm is only related to a user-defined error tolerance. A detailed analysis of such algorithms is presented in [24]. Performance of SDH algorithms can be further improved if certain inherent properties of the simulation system are utilized. It is often observed in MS systems that the atoms are evenly spread out due to existence of chemical bonds and inter-particle forces. Therefore, it is possible to take advantage of the spatial uniformity of the atoms to speed up the computation of SDH. With the spatial distribution of atoms in nodes, we can derive the distribution of distances between any pair of nodes. The entire distribution (i.e., histogram) can thus be obtained by considering all such pairs. Similarly, the locality of atoms in different frames is also utilized to compute approximate SDH very efficiently [25].

Our algorithm performs the same operation on different pairs of regions. This gives us a hint to use parallel processing to further improve performance. General Purpose Computing on Graphics Processing Unit (GPGPU) is a low-cost high performance solution to parallel processing problems. Large number of parallel threads can be created on GPUs, which are executed on multiple cores. Unlike CPUs, the GPU architecture consists of more than one level of memory that can be addressed by the user program - the *Global memory* and the *Shared memory*. The latter is a cache grade memory with extremely low latency. This makes code optimization in GPUs a very challenging task. We developed and optimized GPU versions of above SDH algorithms and achieved dramatic speedup of computation [25].

#### View-based query processing

**Figure 6:**
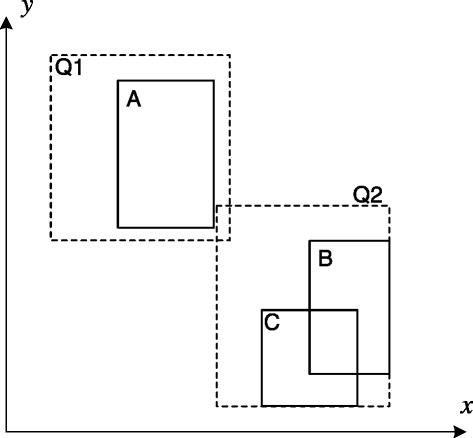
**Two spatial queries (Q1, Q2) and three recorded views (A, B, C) in a 2D space.**

We developed a system-level approach to further improve the response time of analytical queries: *reuse the results of previous queries*. This is a generalization of the caching-based strategy and can be realized by defining a query as a *view* and caching its results (i.e., view materialization). In extreme cases where the same query is re-issued, it can be answered instantly by retrieving the recorded view. A more general situation is: new queries, although not identical to any materialized views, could benefit from one or more such views. For example, the results of the mass center (MC) queries against regions A, B, and C in Figure 6 are stored as views. To compute a newly-issued MC query against region Q1, we could utilize the MC of region A (from the corresponding view), with relevant values cached in TPS tree nodes corresponding to region *Q*1−*A*; to compute a query against Q2, we can first compute the results in region *B*∪*C* (based on materialized views of B and C), and then process the query in region *Q*2−(*B*∪*C*). Clearly, we save the time to compute query against regions A,B, and C from scratch in processing queries Q1 and Q2. As compared to the caching-based strategy, the view-based solution is more efficient since it is not constrained to visiting all tree nodes involved in the query region.

To make the view-based solution work, the main challenge is the *design of query optimization algorithms that take views into account*. Query optimizers of existing DBMSs are not established for our purpose: they focus on views that are built over various base tables [26,27] in the database, often as a result of join operations. On the other hand, a view in our system maps a multidimensional data region to a complex aggregate. Such differences require development of novel techniques to address the following research problems.

##### View representation

The first problem is *how to represent and store recorded views*. Since any view of interest in DCMS describes a certain type of query (e.g., mass center) over a collection of raw data points in a 3D region, we organize the views in data structures similar to the spatial trees used for indexing the data. We call this the *view index*, in which a leaf node has the form of (*BR*,*t*_1_,*t*_2_,*TYPE*,*rprt*) where *BR* (short for Bounding Region, as in R-trees [28]) is a description of the query region of the view, *TYPE* is a variable encoding the query type and parameters (e.g., resolution, maximum, and minimum of an SDH query), *t*_1_ and *t*_2_ refer to the starting and ending frame the query covers, and *rprt* points to the query results of the view. An R*-tree-like structure is designed to organize the view entries based on their BRs. Upon receiving a query, we search the view index using the *BR* value of the new query as the key and retrieve all views that match the type and overlap with the query in their BRs and temporal coverage. The set of views retrieved form the basis for query optimization. We found the cost of maintaining and searching the view index is small due to its tree-based structure and the moderate number of views (as compared to the number of the data points).

##### Cost evaluation of view-based plans

Given a set of matched views, there could be multiple ways (i.e., execution plans) to execute a relevant query. For instance, to compute Q2 in Figure 6, we could either compute the result in region *B*∪*C* and merge it with that of *Q*2−(*B*∪*C*), or merge the results of region *Q*2−*C* with that of *C*, or *Q*2−*B* with *B*. The query optimizer of DCMS should be able to list the different execution plans and choose one with the lowest expected cost. Obviously, those plans that do not involve any views should also be evaluated for comparison. For this purpose, *a cost model* for each query type is designed to quantify the time needed to accomplish a plan. Factors that are considered in the model include: area/volume of the relevant regions involved, expected number of data points in these regions, costs to resolve views with overlapping BRs (e.g., costs to compute the query in *B*∪*C*, which can be used to solve Q2), and existence of indexes. For queries with a small number of execution plans, the decision on which plan to choose can be made by evaluating the costs of all the plans. We are in the process of designing heuristic algorithms to help make decisions with reasonable response time in facing a large number of execution plans.

##### View selection

Storage space is the only cost in maintaining views in DCMS due to the nearly read-only feature of the database. However, this does not mean we can keep as many views as we want (even if enough storage space is available). The reason is that, when the number of views increases, the view index is packed with more and more entries with overlapping BRs. This has two undesirable effects: (1) since the search performance of R-tree-like data structures deteriorates when the nodes have larger overlapping regions, the time needed to retrieve relevant views from the tree increases; (2) the number of relevant views retrieved for a given query will also become larger. As a result, the number of execution plans increases exponentially. These make view-based query processing less attractive as the time used by the query optimizer in query evaluation could grow unboundedly. To that end, we need to discard materialized views from the view index and/or stop materializing new views when the performance gain of view-based query processing reaches zero. A scoring function with the following style is used to determine which views to discard/keep:

$$S = \frac {f} {o} $$

where *f* is the frequency at which the view is utilized for query execution and *o* shows the extent to which the view’s BR overlaps with other materialized views. In case of enforced view selection, a view with lower score will be discarded before one with a higher score. The intuition behind the above formula is to calculate the benefit to cost ratio of view maintenance: views that are frequently used for query processing (i.e., more benefit) and cover a less crowded region (i.e., lower maintenance/query optimization cost) should be kept. Note that storage cost is not included in the scoring function as we believe it is not the bottlenecking factor in our view-based query processing.

### Data compression

Simulation information is stored onto disk frame by frame for further analysis of the system under study. Given large number of particles in the system, a simulation of few micro-seconds can generate terabytes of data. The size of the data poses problems in input/output, storage, and network transfer. Therefore, compression of simulation data is very important. Traditional compression techniques can’t achieve high compression ratio. Size of the data compressed using dictionary-based and statistical methods can still be large. Accessing a small portion of the compressed data requires decompression of entire data set. In addition, corruption of few bits can make the entire data unusable. Techniques that use spatial uniformity of the data, such as space-filling curves [29] can produce better compression ratios. But, all existing methods do not consider temporal locality for further compression. We group several frames together to form a window, and a window is compressed using our technique explained below.

**Figure 7:**
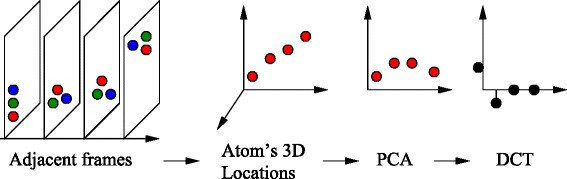
**Steps in our MS data compression algorithm (borrowed from [** 30 **]).**

In our framework (Figure 7), the data is first transformed, using principal component analysis (PCA), from 3D coordinate space to another 3D eigen space, with the dimensions sorted in decreasing importance levels in capturing the variance of the atoms’ movements. In the eigen space, the discrete cosine transform (DCT) is applied to achieve lossy compression across a window of consecutive frames. One major design goal of our framework is that the lossy compression does not affect the results of the analytics that are often executed on MS data. Our technique fusing PCA and DCT enables our framework to: (1) perform balanced compression across all dimensions, (2) control and avoid compression errors and data corruption; and (3) allow more random access to any frame in the data – only a window of frames compressed together is accessed instead of fully decompressing the whole data file.

### Security issues

Preserving data privacy is critical to organizations and research groups that employ external or third party analysts to understand the data, find out interesting patterns, or make new discoveries, but there are concerns on sharing the raw data. Sometimes, scientists have the same concerns over MS data. Privacy can be provided by database management systems through access control mechanisms (ACMs). ACMs limit the data access to users with special privileges, and ACM policies are directly supported by the SQL language. ACMs either restrict or completely grant access to the data. However, third party analysts may not be able to perform the best work without accessing other parts of the data that may depend on the private information. Attempts to provide flexibility ended with differential privacy mechanisms, which also face limitations due to requirements that are difficult to quantify, for both data providers and analysts. Therefore, the problem of preserving privacy from the range of ACMs to differential privacy is inadequately addressed. We performed some fundamental research on this topic within the context of DCMS. In particular, we designed an architecture named security automata model (SAM) to enforce privacy-preserving policies. SAM allows only aggregate queries, as long as privacy is preserved. Once it detects possible risk, differential privacy policy is enforced. It works on basic aggregate queries, liberating data owners from controlling special programs written by the analysts. Sequence of queries from all users in different sessions are monitored to detect the privacy breaches. We integrated this design into DCMS to address the question of how privacy can be defined, enforced, and integrated with existing ACMs using SAM.

## Discussion and evaluation

We have implemented a prototype of DCMS and tested it with real MS datasets and workloads. We used PostgreSQL (version 8.2.6) as our database engine. We extended the current PostgreSQL codebase significantly by adding new data types, the TPS tree index, and various query processing and optimization algorithms. The TPS tree implementation, along with the addition of two new data types (i.e., 3D point and 3D box) was built on top of the SP-GiST [31] package. The query processing algorithms were programmed in following three ways: (1) most one-body functions are implemented as stored procedures using PL/pgSQL – a built-in procedural language provided by PostgreSQL; (2) the more complex multi-body functions are programmed as C functions and integrated into the PostgreSQL query interface; and (3) query processing algorithms related to the TPS tree are directly implemented as part of the PostgreSQL kernel. The data transformation module and data compressor were implemented as programs outside the DBMS. In the remainder of this 2, we report results of selected experiments run on our prototype. Such experiments were conducted on a Mac Xserve with quad-core Intel Xeon CPU running OS X 10.6 operating system (Snow Leopard), the server is connected to a storage array with a 35TB capacity with dual FibreChannel links.

### Efficiency of data retrieval in DCMS

The main goal of this experiment is to show the efficiency of data retrieval in DCMS. Here we show the results of queries against a single MS dataset with 286,000 atoms and 100,000 frames, the total data size of which is about 250 GB. Note that such queries against a single simulation are typical in MS applications as comparing multiple simulations is less popular. This dataset was generated from our previous work to simulate a hydrated DPPC system in NaCl and KCl solutions [23]. For comparison, we sent the same queries against the data analysis toolkit of GROMACS – a mainstream file-based system for MS simulation and data analysis [9]. According to [6], GROMACS has better performance over other popular MS systems therefore represents the state-of-the-art in MS data analysis. We tested the following types of queries: (1) random point access (RDM); (2) single-atom trajectory retrieval (TRJ); (3) frame retrieval (FRM); (4) Range query (RNG); and (5) Nearest neighbor (NN) queries. These queries, as discussed in Section “Analytical queries in DCMS”, form the basis for most high-level data processing tasks in MS. Upon loading the data, PostgreSQL automatically builds a B^+^-tree index on the combination of step number and atom ID. Then we built the TPS tree on the *Atom Location* table for our experiments. For the control experiments using GROMACS, the queries were against the same dataset organized in 400 files, each holding 250 time frames. To achieve fair comparisons, we also used a grid-based spatial index in GROMACS [9] for the RNG and NN queries.

The main results of this experiment are shown in Table 2 where each number is the average of five experiments of the same query with randomly generated parameters. Clearly, the query performance is much better in DCMS, especially when we built the TPS tree. In fact, it achieves a speedup of 1-5 orders of magnitude over GROMACS. This shows the combined effects of record-based (rather than file-based) I/O and indexes. By the latter, we can directly visit the pages that hold relevant data records while we have to search through large files in the file-based solution. An interesting thing is that TRJ processing in DCMS, although faster than in GROMACS, still requires very long time when the TPS tree is not used. This shows that the existing indexes in PostgreSQL are not suitable for such queries. Apparently, all files are scanned in processing TRJ queries in GROMACS. Although indexes were used in GROMACS in processing the RNG and NN queries, we still observe a performance boost of 2-3 orders of magnitude in DCMS. The spatial index of GROMACS does not help at all in processing the RDM, TRJ, and FRM queries. In summary, the above results demonstrate significant improvement in data access speed by using DCMS for MS data processing.

**Table 2:** **Query processing time (in seconds) in database-centric and file-based MS analysis**

**System**	**Queries**				
	**RDM**	**TRJ**	**FRM**	**RNG**	**NN**
DCMS + TPS	0.0008	13.4	2.56	0.073*	0.029
DCMS	0.069	6239	2.48	0.122*	0.198
GROMACS	45.0	16410	52.5	8.49	16.8

*time depends on the query range

### SDH Computation results

**Figure 8:**
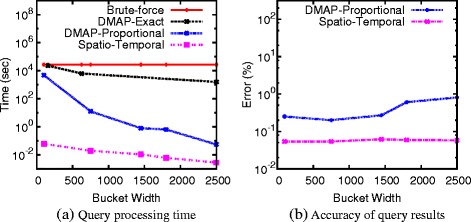
**Performance of different SDH algorithms under different histogram resolutions.**

Synthetic data and real data generated from a collagen fiber simulation with 890,000 atoms are used for testing the performance of our SDH algorithms, with results summarized in Figure 8. Comparing with the brute force method, the density-based algorithm is about ten times faster under the larger bucket width. Once we consider the approximate algorithm, performance boost quickly reaches 2-3 orders of magnitude. Our ultimate solution that takes advantage of spatio-temporal locality of data further widens this gap - it is 3-4 orders of magnitude more efficient than the brute-force method. On the other hand, the errors generated from the approximate algorithms are very small (Figure 8(b)) - they are kept at the level of 1% for the density-based algorithm and go down to about 0.05% for the algorithm that considers spatio-temporal locality. Note that, the accuracy of our algorithms is not only small in practice, it is also guaranteed by a bound. Such details and other experimental results can be found in our previous publications [23**,**^24^].

We also implemented and experimented various algorithms based on GPUs. We developed our program under Nvidia’s CUDA [32] parallel computing framework. The results of our implementation of the brute-force algorithm are shown in Table 3. Experiments were run on an Nvidia GTX570 GPU and a 2.66 GHz Intel Xeon CPU. We were able to achieve a speedup of up to 40 × against the CPU version while only using the Global Memory (GM) in the GPU algorithm. More efforts put into developing a Shared Memory (SM) based program yielded amazing returns of a 258 × improvement. The GPU version of the algorithm using spatial locality was also experimented, and reported a 20 × improvement (see [25] for more details). Based on our findings we believe that the GPUs are very powerful in processing complex analytics related to MS.

**Table 3:** **Running time (seconds) of brute-force SDH method on GPU**

**System**	**GPU-time**		**CPU**	**GPU-speedup**	
**Size**	**SM**	**GM**	**Time**	**SM**	**GM**
100,000	1.86	11.15	424.7	228	38
300,000	15.24	96.6	3812.2	250	39
800, 000	105.15	677.1	27142	258	40
8,000,000	3 hrs.	–	> 27 days	> 216	–

### Data compression results

**Figure 9:**
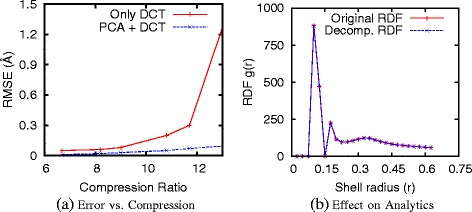
**Performance of our MS data compression method: (a) compression ratio and error; (b) effects on radial distribution function (RDF).** More details can be found in [30].

We used the same data mentioned in Section “Efficiency of data retrieval in DCMS” to test the MS data compression method. Specifically, about 600 frames captured during the aforementioned simulation were used for our experiments. The following properties of atoms were stored in the file: x, y, and z coordinates, charge and mass measured. To measure the quality of compressed data, we use root mean square error (RMSE) to quantify the difference between the original data and the decompressed data. Figure 9(a) summarizes the main results. It can be seen that high compression ratio was achieved along with low RMSE between original and decompressed data. With very little loss of information, we achieved a compression ratio of at least 12 in such experiments. The effect of compression on the accuracy of analytical queries is also small (Figure 9(b)): for a shell resolution of 0.025Å, the difference between the RDFs of the original and decompressed data is negligible.

## Other related work

Traditionally, database systems are mainly designed for commercial data and applications. In recent years, the scientific community has also adopted database technology in processing scientific data. However, scientific data are different from commercial data in that: (1) the volume of scientific data can be orders of magnitude larger; (2) data are often multidimensional and continuous; and (3) queries against scientific data are more complex. The above differences bring significant challenges to system design in scientific databases.

In summary, scientific database research fall into the following three types. The first is to build databases on top of out-of-box DBMS products, as seen in the following examples: GenBank (http://www.ncbi.nlm.nih.gov/Genbank) provides public access to about 80 million gene sequences; the Sloan Digital Sky Survey [33] enables astronomers to explore millions of objects in the sky; and the PeriScope [34] project explores declarative queries against biological sequence data. The second type focuses on extending the kernel functionalities of DBMSs to meet challenges in scientific data management. This includes work that deals with query language [35], data storage [36,37], data compression [38,39], index design [40], I/O scheduling [41], and data provenance [42]. The last type takes a more aggressive path by designing new DBMS architectures and building the DBMS from scratch. Most efforts along this direction happened in the past few years [43-46]. The SciDB project advocates a new data model (i.e., the multidimensional array model) for scientific domains and releases a prototype that enables parallel processing of data in a highly distributed environment. Clearly, our strategy of building DCMS falls into the second category.

## Conclusions

Despite the importance of MS as a major research tool in many scientific and engineering fields, there is a lack of systems for effective data management. To that end, we developed a unified Information Integration and Informatics framework named Database-centric Molecular Simulations (DCMS). DCMS is designed to store simulation data collected from various sources, provide standard APIs for efficient data retrieval and analysis, and allow global data access to the research community. This framework is also a portal for registering well–accepted queries that in turn serve as building blocks for more complex high–level analytical programs. Users can develop these high–level programs into applications such as, applications that grant easy access of their data to experimentalists, visualize data, and provide feedback which can be used in the steering of MS. A fundamental component of the DCMS system is a relational database, which allows scientists to concentrate on developing high-level analytical tools using declarative query languages while passing the low-level details (e.g., primary query processing, data storage, basic access control) to DCMS. One of the most serious problems in existing MS systems is the low efficiency of data access and query processing. The unique query patterns of MS applications impose interesting challenges and also provide abundant optimization opportunities to DCMS design. To meet such challenges, we augmented an open-source DBMS with novel data structures and algorithms for efficient data retrieval and query processing. We focused on creative indexing and data organization techniques, query processing algorithms and optimization strategies. The DCMS system was also used as a platform to evaluate data compression algorithms specifically designed for MS data that can significantly reduce the size of the data.

Immediate work within DCMS will be focused on sharing computations (especially I/O operations) among different queries. Unlike traditional database applications, MS analysis normally centers around a small number of analytical queries (Table 1). Therefore, we can pro-actively run all relevant analytics at the time when the data is being loaded to DCMS. The advantage of this strategy is that only one I/O stream is needed - we have shown earlier that I/O can be the bottleneck in handling typical MS workloads. This requires us to modify the DBMS kernel to implement a master query processing algorithm to replace the ones dealing with individual queries. On the query processing side, utilization of other parallel hardware such as multi-core CPUs and FPGAs is definitely worth more efforts. Our current design of DCMS focuses on a single-node environment, deployment of DCMS on modern data processing platforms in a highly distributed environment (e.g., a computing cloud) will be an obvious direction for our future exploration.
